# Influence of short periods of increased water temperature on species composition and photosynthetic activity in the Baltic periphyton communities

**DOI:** 10.2478/s11756-018-0122-6

**Published:** 2018-09-13

**Authors:** Filip Pniewski, Zuzanna Sylwestrzak

**Affiliations:** Institute of Oceanography, University of Gdańsk, Al. Piłsudskiego 46, 81-378 Gdynia, Poland

**Keywords:** Periphyton, Chlorophyll fluorescence, Diatoms, Temperature, Baltic Sea

## Abstract

Periphyton plays a vital ecological role in shallow, well-lit ecosystems which are vulnerable to rapidly changing environmental conditions, including raising temperature due to global warming. Nevertheless, little is known on the effect of increased temperatures on the taxonomic structure and functioning of periphytic communities. In this study, the influence of short-term temperature increase on the species composition and photosynthetic activity of the Baltic periphytic communities was investigated. The collected communities were exposed to increased temperature of 23 °C (ca. 4 °C above the summer average) for 72 h. After this time, species composition of the communities was studied under light microscope and their photosynthetic performance was evaluated using PAM fluorometry. Results showed that the biomass of cyanobacteria slightly increased. There were significant changes in the abundance of diatom species, among which *Fragilaria fasciculata* and *Navicula ramosissima*, were negatively affected by the elevated temperature and their cell number significantly decreased, whereas, *Diatoma moniliformis* and *N. perminuta* were stimulated by the increased temperature. Additionally, a shift towards higher abundance of smaller taxa was also observed. The higher quantum yield of photosystem II (PSII) (higher *Φ*PSII) accompanied by the lower value of non-photochemical quenching (NPQ) observed in communities kept at 23 °C showed more efficient photosynthesis. This was further confirmed by the changes in rapid light curves (higher photosynthetic capacity, rETR_max_, and photoacclimation index, E_k_). The obtained data constitute evidence that short periods of increased temperature significantly affect the structure and functioning of the Baltic periphyton.

## Introduction

Periphyton assemblages (algal biofilms) can be found on a variety of substrata submerged in water. They can thrive on a solid surface-water interface by excreting extracellular polymeric substances. Mature periphytic assemblages have three-dimensional structure, including taxa of different growth forms (Tuji 2000; Gulzar et al. 2017). Periphyton can be responsible for majority of primary production especially in shallow well-lit habitats, in such aquatic environments as lakes, rivers, coastal waters etc. (Dodds et al. 1999). It is also an important source of food for invertebrates (Gulzar et al. 2017). Interacting with the surrounding environment periphyton affects a concentration of nutrients and provides oxygen (Gaiser 2009). Furthermore, periphyton, due to its short life cycle, quickly responds to pollution and changes in environmental conditions, and thus it is often used as an indicator of water quality (Gulzar et al. 2017).

Nowadays, the environment is undergoing a dramatic change due to global warming, including not only continually increasing temperatures but also the frequency and intensity of climate extreme phenomena such as heat waves (Vieira et al. 2013). Recent studies proved temperature increase in the Baltic and have shown that in summer the average temperatures of ca. 19 °C in the Southern Baltic are recorded. However, occasionally short periods (several days) of temperatures above 23 °C can be also observed (http://www.satbaltyk.pl) (Siegel et al. 2006; Bradtke et al. 2010; Woźniak et al. 2011; Rak and Wieczorek 2012; Stramska and Białogrodzka 2015). Despite the ecological importance of periphyton, little is known on the influence of increased temperature on its structure and functioning. The analyses of diatom dominated benthic communities from intertidal mudflats have shown that transient higher temperatures stimulated its photosynthesis, whereas, prolonged high temperature exposer led to the biomass decrease, photosynthesis impairment and changes in species composition promoting growth of cyanobacteria (Hicks et al. 2011; Vieira et al. 2013; Cartaxana et al. 2015). In subtidal systems a shift towards heterotrophic benthic communities was also recorded (Hancke and Guld 2004). Such strong effects observed for benthic microalgae suggest that periphyton may also undergo significant temperature-driven changes.

Thus, the aim of this study was to investigate the influence of short-term temperature increase on the structure and photosynthetic activity of the Baltic periphytic communities. In this study, species composition of periphyton kept at control (18 °C) and elevated (23 °C) temperatures was investigated and their photosynthetic activity was assessed by means of variable chlorophyll fluorescence.

## Materials and methods

### Sampling site, microalgal suspension preparation and experimental setup

Periphytic communities were collected from outdoors cultivation panels (Sylwestrzak et al. 2018) submerged at the depth of ca. 2 m in the littoral zone of the Gulf of Gdańsk, ca. 300 m from the shore near Sopot (the Baltic, 54°26′N, 18°33′E). Periphyton was grown on glass slides (2 cm × 8 cm) for a week in July 2015. After the exposition period, panels were collected and transported to the laboratory where microalgae were gently scrapped from the glasses and resuspended in the Baltic water of salinity ca. 8 collected at the sampling site and filtered through the sterile membrane filters with pore size of 0.45 μm (Millipore Sterile HA Filters). To prepare periphyton cultures 100 ml Erlenmeyer flasks were filled with 50 ml of microalgal suspension. Subsequently, they were kept under constant irradiance (provided by fluorescent lamps Phillips 40 W) and temperature conditions, i.e. 60 μmol photons m^−2^ s^−1^ (as measured by a quantum meter LiCor LI-198 with cosine collector) in 16 h light:8 h dark cycles and 18 °C (a water temperature measured during the sampling), for three days. After acclimatization period, on the starting day of the experiment species composition, photosynthetic pigment content and photosynthetic activity was analyzed in three randomly chosen out of six periphyton cultures. Subsequently, the cultures were divided into two groups; one of them was moved to higher temperature, i.e. 23 °C, whereas the second one was further kept at 18 °C. Both groups of cultures were incubated under experimental conditions for another 3-day period. During this time light conditions remained the same. All measurements were carried out in triplicates.

### The study of periphyton species composition

Species composition of each replicate was studied under light microscope Nikon 80i equipped with DS-U2 camera using 40× objective. In order to establish the relative abundance of cyanobacteria and microalgae at least 300 cells were counted. The biovolume of counted species was calculated according to Olenina et al. (2006). Subsequently, the biomass (wet weight) of each taxonomic group was derived based on an assumption of a plasma density of 1 g cm^−3^ across all taxa (HELCOM 2013). To study diatoms permanent slides were prepared; periphyton samples were treated with hydrogen peroxide at 30–90 °C for 3 – 6 h, then rinsed with water, mounted in Naphrax (Battarbee 1986) and analyzed with the same microscope under 100× oil immersion objective, counting at least 300 frustules.

### Photosynthetic pigment analyses

Ten ml aliquots of periphyton samples were filtered through GF/C Whatman glass filters (25 mm diameter) under low vacuum then frozen and stored at −20 °C until further processing. Pigments were extracted using 90% acetone as described in Pniewski et al. (2015). Subsequently, pigments were analyzed using Waters HPLC system equipped with Water 2998 Photodiode Array Detector. Pigments were separated using reverse phase chromatography (RP-HPLC) (250 mm × 4.6 mm LiChrospher®100 RP-100 endcapped column) following optimized protocol by Stoń and Kosakowska (2002). The HPLC system was calibrated using pigment standards purchased from The International Agency for 14C Determination DHI Institute for Water and Environment in Denmark. Pigments were identified from their retention times and absorbance spectra, and quantified according to the procedure provided by Mantoura and Repeta (1997).

### Measurements of chlorophyll a fluorescence

Measurements of chlorophyll *a* fluorescence were carried out using a computer-operated Fluorescence Monitoring System (FMS1; Hansatech, Norfolk, UK). The device provides amber light with emission maximum at 594 nm to excite fluorescence and a PIN-photodiode at wavelengths beyond 700 nm to detect it. An integral halogen lamp (8 V/20 W) provides actinic as well as saturating irradiance. Light was measured with a Li-Cor LI-189 quantum-meter with a cosine collector. All measurements were made with 5.5-mm-diameter Fiberoptic kept perpendicularly to the biofilm at the constant distance of 4 mm.

Similarly as with pigment analysis, five ml aliquots of pheriphyton cultures were filtered through GF/C Whatman glass filters (6 mm diameter) under low vacuum and placed in the thermoregulated DW2 chamber. Periphyton samples were dark-adapted for 15 min to measure the maximum quantum yield (Fv/Fm) of photosystem II (PSII) (Genty et al. 1989). Subsequently, samples were illuminated with the actinic light of 60 μmol photons m^−2^ s^−1^ for 8 min in order to reach a steady-state and the effective quantum yield of PSII in the light-adapted state (*Φ*PSII) and non-photochemical quenching (NPQ) were measured. After the light period, samples were exposed to 9 increasing light intensities from 10 to 1280 μmol photons m^−2^ s^−1^ in order to construct rapid light curve (RLCs) with light duration step of 10 s and at each light step the relative electron transport rate (rETR) was calculated (Ralph and Gademann 2005; Lefebvre et al. 2011). To quantitatively compare RLC curves empirical data were mathematically fitted to the model of Jassby and Platt (1976) and photosynthetic parameters, i.e. the maximum relative electron transport rate (rETR_max_), the initial slope of the rETR vs. E response curve (α), the light saturation index (E_k_) were estimated (Sakshaug et al. 1997).

### Statistics

To compare the mean values of the analyzed parameters Student t-test was used. First, the means calculated on the starting and the last day of the experiment for cultures kept at 18 °C were compared to track statistically significant changes occurring due to the incubation period itself. Subsequently, the mean values of all parameters calculated for periphytn cultures maintained at different temperatures, i.e. 18 and 23 °C, on the third day of the incubation period were compared to assess the effect of temperature on the studied assemblages. All statistical analyses were performed using Statistica 10 (StatSoft Inc., USA).

## Results

After the 3-day acclimatization period the biomass of the communities, as measured by the chlorophyll *a* (Chl*a*) concentration, stabilized (ca. 0.36 μg Chl*a* ml^−1^). There was no change in community biomass (0.36 ± 0.02 μg Chl*a* ml^−1^) at the lower temperature (18 °C) on the third day of cultivation period compared to the starting day. In communities exposed to the higher temperature (23 °C) Chl*a* concentration only slightly decreased (0.30 ± 0.06 μg Chl*a* ml^−1^) compared to the communities maintained at the lower temperature (Student t-test; *p* > 0.05). Furthermore, there were slight, but statistically not significant, changes regarding species and photosynthetic pigment composition as well as parameters estimated to describe periphyton photosynthetic activity in communities kept at the lower temperature compering the starting (K 18 °C) and the last day of the experiment (E 18 °C). Clear differences emerged only when the communities from different temperature treatments were compared at the end of the experiment (Student t-test; *p* < 0.05).

**Fig. 1:**
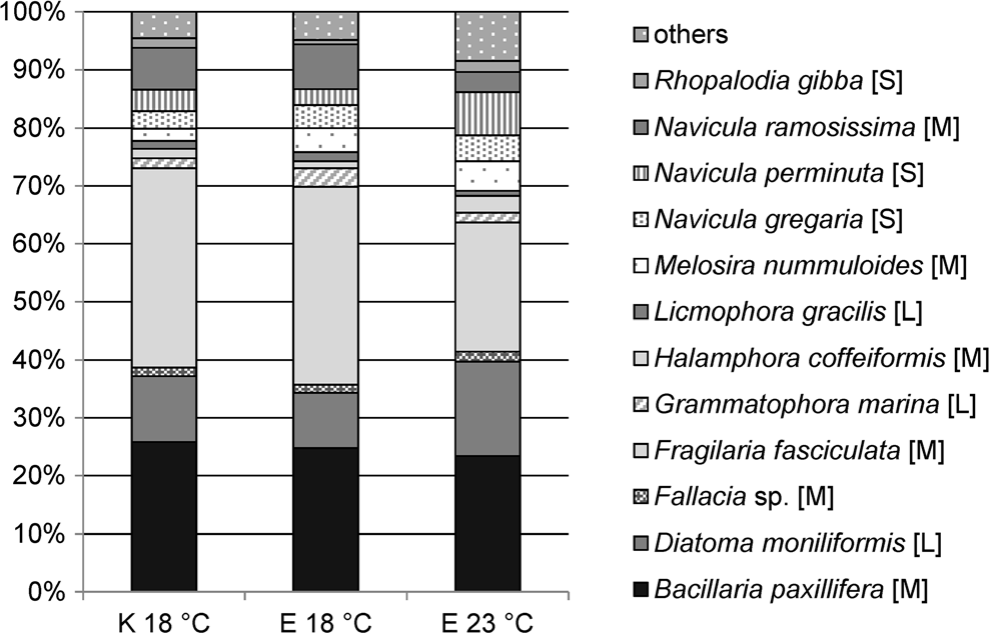
Diatoms species composition of the periphyton communities; K 18 °C – the initial community observed on the starting day of the experiment, E 18 °C – the community developed after 3-day incubation period at the temperature of 18 °C, E 23 °C – the community developed after 3-day incubation period at the temperature of 23 °C. Letters denote the size class of species; S – small (<1000 μm^3^), M – medium (1000–5000 μm^3^), B – large (>5000 μm^3^)

Diatoms dominated in the studied assemblages contributing more than 91% to their biomass. The remaining biomass was due to the cyanobacterial input and with the increase in temperature the ca. 2.5-fold increase in cyanobacterial biomass form 3.8% at 18 °C to 8.4% at 23 °C was observed. Different temperature conditions altered the composition and structure of the assemblages. The total number of 29 species was identified in the samples. Under each experimental treatment species richness was 20–28. Among the identified species cyanobacteria were represented by three genera, i.e. *Anabaena*, *Merismopedia* and *Spirulina*. The remaining species were diatoms of which twelve species constituted more than 90% of the cell count (Fig. 1). The diatom composition showed that at both temperatures assemblages were dominated by species of medium size (1000–5000 μm^3^), 84 and 77.5% at 18 °C and 23 °C, respectively, and at the elevated temperature the proportion of smaller diatoms (with volume below 1000 μm^3^) significantly increased (by ca. 7%) up to 17.7% (Student t-test; *p* > 0.05). Diatoms of bigger size (>5000 μm^3^) occurred less frequently and their abundance remained relatively constant. The most abundant species were: *Bacillaria paxillifera* (O.F.Müller) T.Marsson 1901, *Diatoma moniliformis* (Kützing) D.M.Williams 2012, *Frgailaria fasciculata* (C.Agardh) Lange-Bertalot 1980, *Navicula perminuta* Grunow in Van Heurck 1880, *N. ramosissima* (C.Agardh) Cleve 1895. Among them, the diatom *B. paxillifer* was not affected by temperature and its abundance remained stable (ca. 24%). Two species, i.e. *F. fasciculata* and *N. ramosissima*, were negatively affected by the elevated temperature and their cell number significantly decreased (Student t-test, *p* < 0.05), whereas *D. moniliformis* and *N. perminuta* were stimulated by temperature and their amount increased (Student t-test, *p* < 0.05).

**Fig. 2:**
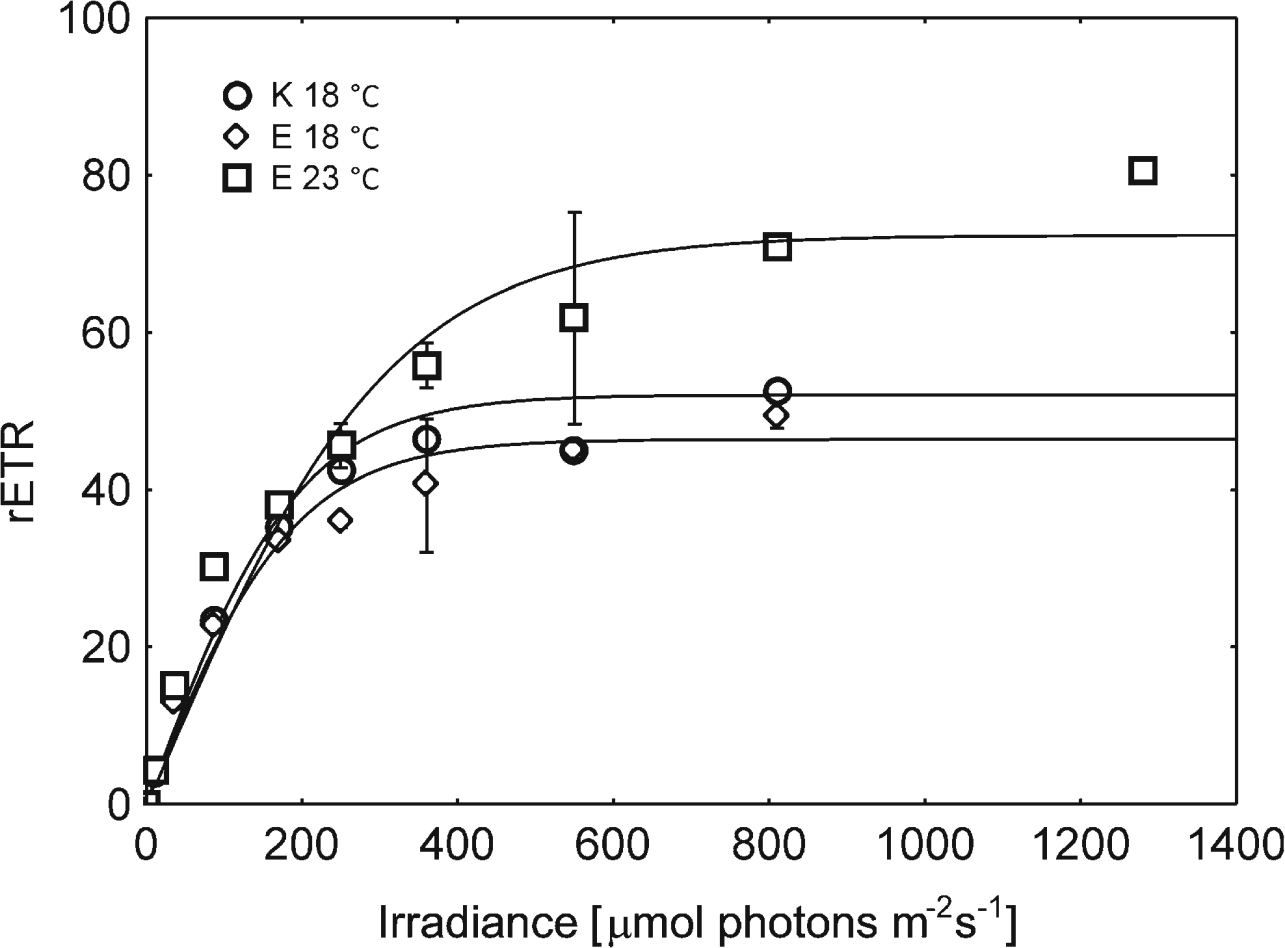
Temperature-driven changes in the shape of RLCs (rapid light curves) measured for the periphyton communities; K 18 °C – the initial community observed on the starting day of the experiment, E 18 °C – the community developed after 3-day incubation period at the temperature of 18 °C, E 23 °C – the community developed after 3-day incubation period at the temperature of 23 °C

**Fig. 3:**
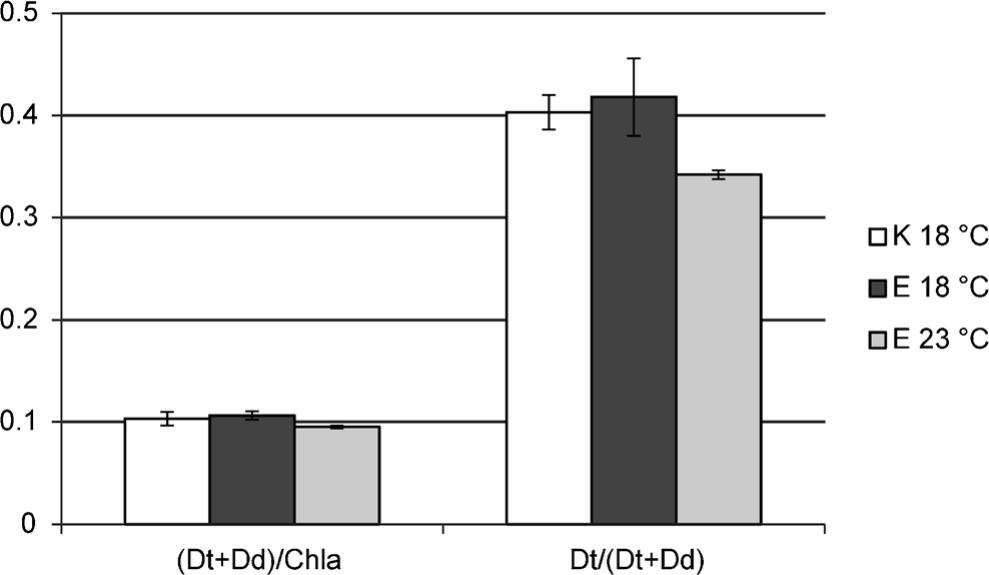
Temperature-driven changes of the xanthophyll cycle pigments in the periphyton communities; K 18 °C – the initial community observed on the starting day of the experiment, E 18 °C – the community developed after 3-day incubation period at the temperature of 18 °C, E 23 °C – the community developed after 3-day incubation period at the temperature of 23 °C; Dd – diadinoxanthin, Dt - diatoxanthin

Regarding the fact that the changes in the species composition of communities kept at the lower temperature (18 °C) throughout the experiment were strongly limited, there were no statistically significant changes in their photosynthetic performance also (Fig. 2, Table 1). Comparing lower and higher temperature treatments it was shown that Fv/Fm changes were not statistically significant. Samples kept at the higher temperature showed higher values of the quantum yield of PSII (*Φ*PSII), whereas in case of the non-photochemical quenching the reverse pattern was observed and its 2-fold decline was recorded. The analysis of RLCs showed that higher temperature increased periphyton photosynthetic activity (Fig. 2, Table 1). The rETR_max_ values increased by ca. 34%, whereas *α* declined by ca. 5%. The behavior of both aforementioned parameters determined the change in the E_k_ value causing its increase at higher temperature conditions reaching 272 μmol photons m^−2^ s^−1^. Changes in variable fluorescence, and by extension in fluorescence parameters and RLCs, are dependent on the activity of xanthophyll cycles. The xanthophyll pool ((Dt + Dd)/Chl*a*) varied slightly during the experiment; at the higher temperature (E 23 °C) (Dt + Dd)/Chl*a* decreased by ca. 10% (Student t-test, *p* < 0.05) compared to the lower one (E 18 °C), whereas the de-epoxidation state of xanthophylls (Dt/Dt + Dd) decreased by ca. 20% (Student t-test, *p* < 0.05) (Fig. 3).

**Table 1:** Fluorescence parameters measured for the periphyton communities; K 18 °C – the initial community observed on the starting day of the experiment, E 18 °C – the community developed after 3-day incubation period at the temperature of 18 °C, E 23 °C – the community developed after 3-day incubation period at the temperature of 23 °C. Fv/Fm – the maximum quantum yield of PSII, *Φ*PSII – the quantum yield of PSII, NPQ – non-photochemical quenching, *α* – the initial part of RLCs (rapid light curves), rETR_max_ – the maximum relative electron transport rate and E_k_ – the index of photoacclimation

	E 18 °C	E 23 °C	
	Mean ± SE	Mean ± SE	P*
Fv/Fm	0.445 ± 0.007	0.476 ± 0.014	0.121
*Φ*PSII	0.293 ± 0.010	0.372 ± 0.009	**0.004**
NPQ	0.45 ± 0.02	0.21 ± 0.05	**0.012**
*α*	0.276 ± 0.017	0.262 ± 0.019	0.388
rETR_max_	46.8 ± 5.7	70.7 ± 7.4	**0.011**
E_k_	170 ± 22	272 ± 48	**0.028**

* Statistically significant differences were shown for *P*<0.05 in bold font

## Discussion

Elevated temperature affected the composition of the studied communities, with diatoms remaining the dominant taxonomic group. The relative abundance of cyanobacteria increased. Similar observations were reported by Cartaxana et al. (2015) who investigated the microphytobenthos community of the Tages estuary. Previous studies have proved that cyanobacteria can be favored under higher temperature conditions (e.g. Latała and Misiewicz 2000; Van der Grinten et al. 2005). On the other hand, Piggott et al. (2015) reported that the raised temperature had no positive effect on cyanobacteria in periphyton communities. Furthermore, diatom communities were also affected by increased temperature. In this study, there was no dramatic shift in the community structure; nevertheless, the relative abundance of eight species was significantly affected (Fig. 1.), including two *Navicula* species, i.e. *N. perminuta* and *N. ramosissima*. Laboratory experiments showed that the former species grew well within a wide temperature range reaching high cell number at 15–25 °C. Furthermore, the higher the light intensity (up to 150 μmol photons m^−2^ s^−1^) was the higher the number of cells was observed (Biskup, unpublished). The latter one, on the other hand, was found in the Baltic proper to be forming large colonies under ice cover (Snoeijs and Kautsky 1989), which may suggest its affinity for lower temperatures. Such divers responses of individual species to elevated water temperatures were further reflected in the proportions of the diatom size classes. The amount of large and medium taxa declined in favor of small ones. Overall, there were no negative effects at the community level, while the responses to elevated temperature were species-specific, in agreement with Piggott et al. (2015).

The elevated temperature clearly increased photosynthetic activity of algal communities. Fluorescence measurements showed that when acclimated to culturing light conditions (constant irradiance of 60 μmol photons m^−2^ s^−1^) the steady-state quantum yield of PSII (*Φ*PSII) reached higher values at higher temperature (Table 1). *Φ*PSII provides information on the proportion of the light absorbed by chlorophyll associated with PSII and used for photochemistry (Maxwell and Johnson 2000). Temperature changes may alter concentration and activity of the electron transport chain as well as Calvin-Benson cycle enzymes (most notably RUBISCO) directly affecting *Φ*PSII (Davison 1991). Therefore, its increase under higher temperature conditions indicated an efficient use of the absorbed photons, and thus higher overall photosynthetic capacity of periphyton.

Furthermore, the higher *Φ*PSII values corresponded with the decline of NPQ (Table 1). Non-photochemical quenching is a photoprotective mechanism which enables safe dissipation of the excessive light energy through the operation of the xanthophyll cycle; in diatoms it involves a reversible conversion of diadinoxanthin into photoprotecive diatoxanthin under high light conditions (Serôdio et al. 2005). In this study, an effective use of absorbed light, as shown by higher *Φ*PSII, lowered the necessity for the effective energy dissipation. This is further supported by the changes in the photosynthetic pigments composition. The de-epoxidation state of xanthophylls (Dt/Dt + Dd) indicated a lower relative amount of diatoxanthin (Fig. 3), explaining lower capacity of periphyton for NPQ build-up under higher temperature (van Leeuwe et al. 2011).

The effective adjustment of the photosynthetic activity of periphyton was also confirmed by RLCs variations. Under limiting light intensities, photosynthetic efficiency of studied communities did not differ significantly as seen from the similar *α* values (Table 1, Fig. 2), similarly to Vieira et al. (2013). Changes in the *α* values are mainly controlled by light conditions which affect photosynthetic pigments’ composition, concentration and packaging, while temperature plays less important role (Henley 1993). With further increase in light intensity, ETR values also increased reaching the maximum value (ETR_max_). The saturated part of the light curves is mainly controlled by the carbon metabolism which is an enzyme mediated process (Davison 1991), thus under higher temperature it may operate more rapidly, subsequently leading to the higher ETR_max_ values (Kirk 1996). The light saturation index E_k_ reflects the optimum light intensity at which microalgae balance the light absorption and its usage and thus E_k_ is considered to be an indicator of their photoacclimation status (Henley 1993; Serôdio et al. 2006). Since *α* values remained constant at both applied temperatures the observed E_k_ changes were the result of the ETR_max_ values increase, in congruence with Salleh and McMinn (2011).

Previous studies showed that temperature may differently affect benthic microalgal communities and its influence may be modified by other factors. Longer exposure of microphtobenthos to elevated temperatures (4–6 °C above seasonal average) often had detrimental effects leading to the decrease in assemblage biomass and causing a shift in the community structure (e.g. Defew et al. 2004; Hicks et al. 2011; Cartaxana et al. 2015; Piggott et al. 2015). The results of this study showed that even short-term increase in water temperature may induce small but still significant changes in the structure of the community (by promoting species preferring higher temperatures and reducing those less resistant to heat stress). Such changes may be facilitated by species-specific photosynthetic responses of dominant species (e.g. Salleh and McMinn 2011). Furthermore, it may be also speculated that several consecutive short-term periods of higher temperature may have even more profound effects, overall leading to much more pronounced changes in community species composition. Gradually accumulated over longer period of time, small changes in the primary producers communities could finally elicit effects observed at higher-trophic levels (Armitage and Fogg 2004; Shurin et al. 2012).

## References

[CR1] Armitage AR, Fogg P (2004). Upward cascading effects of nutrients: shifts in a benthic microargal community and a negative herbivore response. Community Ecol.

[CR2] Battarbee RW, Berglund BE (1986). Diatom analysis. Handbook of holocene palaeoecology and palaeohydrology.

[CR3] Bradtke K, Herman A, Urbański J (2010). Spatial and interannual variations of seasonal sea surface temperature patterns in the Baltic Sea. Oceanologia.

[CR4] Cartaxana P, Vieira S, Ribeiro L, Rocha RJM, Cruz S, Calado R, da Silva JM (2015). Effects of elevated temperature and CO_2_ on intertidal microphytobenthos. BMC Ecol.

[CR5] Davison IR (1991). Environmental effects on algal photosynthesis: temperature. J Phycol.

[CR6] Defew EC, Perkins RG, Paterson DM (2004). The influence of light and temperature interactions on a natural estuarine microphytobenthic assemblage. Biofilms.

[CR7] Dodds WK, Biggs BJF, Lowe RL (1999). Photosynthesis-irradiance patterns in benthic microalgae: variations as a function of assemblage thickness and community structure. J Phycol.

[CR8] Gaiser Evelyn (2009). Periphyton as an indicator of restoration in the Florida Everglades. Ecological Indicators.

[CR9] Genty B, Briantais J-M, Baker NR (1989). The relationship between the quantum yield of photosynthetic electron transport and quenching of chlorophyll fluorescence. Biochim Biophys Acta.

[CR10] Gulzar A, Mehmood MA, Chaudhary R (2017) Stream periphyton community: a brief review on ecological importance and regulation. IJAPSA. 10.22623/ijapsa.2017.3100.utf4v

[CR11] Hancke K, Guld RN (2004). Temperature effects on respiration and photosynthesis in three diatom-dominated benthic communities. Aquat Microb Ecol.

[CR12] HELCOM (2013) Manual for marine monitoring in the COMBINE programme

[CR13] Henley WJ (1993). Measurement and interpretation of photosynthetic light-response curves in algae in the context of photoinhibition and diel change. J Phycol.

[CR14] Hicks Natalie, Bulling Mark T, Solan Martin, Raffaelli Dave, White Piran CL, Paterson David M (2011). Impact of biodiversity-climate futures on primary production and metabolism in a model benthic estuarine system. BMC Ecology.

[CR15] Jassby AD, Platt T (1976). Mathematical formulation of the relationship between photosynthesis and light for phytoplankton. Limnol Oceanogr.

[CR16] Kirk JTO (1996). Light and photosynthesis in aquatic ecosystems.

[CR17] Latała A, Misiewicz S (2000). Effects of light, temperature and salinity on the growth and chlorophyll-a content of Baltic cyanobacterium *Phormidium* sp. Algol Stud.

[CR18] Lefebvre S, Mouget J-L, Lavaud J (2011). Duration of rapid light curves for determining the photosynthetic activity of microphytobenthos biofilm *in situ*. Aquat Bot.

[CR19] Mantoura RFC, Repeta DJ, Jeffrey SW, RFC M, Wright SW (1997). Calibration methods for HPLC. Phytoplankton pigments in oceanography: guidelines to modern methods.

[CR20] Maxwell K, Johnson GN (2000). Chlorophyll fluorescence – a practical guide. J Exp Bot.

[CR21] Olenina I, Hajdu S, Andersson A, Edler L, Andersson A, Wasmund N, Busch S, Göbel J, Gromisz S, Huseby S, Huttunen M, Jaanus A, Kokkonen P, Ledaine I, Niemkiewicz E (2006). Biovolumes and size-classes of phytoplankton in the Baltic Sea. Balt Sea Environ Proc.

[CR22] Piggott JJ, Salis RK, Lear G, Townsend CR, Matthaei CD (2015). Climate warming and agricultural stressors interact to determine stream periphyton community composition. Glob Chang Biol.

[CR23] Pniewski FF, Biskup P, Bubak I, Richard P, Latała A, Blanchard G (2015). Photo-regulation in microphytobenthos from intertidal mudflats and non-tidal coastal shallows. Estuar Coast Shelf Sci.

[CR24] Rak D, Wieczorek P (2012). Variability of temperature and salinity over the last decade in selected regions of the southern Baltic Sea. Oceanologia.

[CR25] Ralph PJ, Gademann R (2005). Rapid light curves: a powerful tool to assess photosynthetic activity. Aquat Bot.

[CR26] Sakshaug E, Bricaud A, Dandonneau Y, Falkowski PG, Kiefer DA, Legendre L, Morel A, Parslow J, Takahashi M (1997). Parameters of photosynthesis: definitions theory and interpretation of results. J Plankton Res.

[CR27] Salleh S, McMinn A (2011). The effects of temperature on the photosynthetic parameters and recovery of two temperate benthic microalgae *Amphora* cf. *coffeaeformis* and *Cocconeis* cf. *sublittoralis* (Bacillariophyceae). J Phycol.

[CR28] Serôdio J, Cruz S, Vieira S, Brotas V (2005). Non-photochemical quenching of chlorophyll fluorescence and operation of the xanthophyll cycle in estuarine microphytobenthos. J Exp Mar Biol Ecol.

[CR29] Serôdio J, Vieira S, Cruz S, Coelho H (2006). Rapid light-response curves of chlorophyll fluorescence in microalgae: relationship to steady-state light curves and non-photochemical quenching in benthic diatom-dominated assemblages. Photosynth Res.

[CR30] Shurin JB, Calsen JL, Greig HS, Kratina P, Thompson PL (2012). Warming shits top-down and bottom-up control of pond food web structure and function. Phil Trans R Soc B.

[CR31] Siegel H, Gerth M, Tschersich G (2006). Sea surface temperature development of the Baltic Sea in the period 1990–2004. Oceanologia.

[CR32] Snoeijs P, Kautsky U (1989). Effects of ice-break on the structure and dynamics of a benthic diatom community in the northern Baltic Sea. Bot Mar.

[CR33] Stoń J, Kosakowska A (2002). Phytoplankton pigments designation – an application of RP-HPLC in qualitative and quantitative analysis. J Appl Phycol.

[CR34] Stramska M, Białogrodzka J (2015). Spatial and temporal variability of sea surface temperature in the Baltic Sea based on 32-years (1982-2013) of satellite data. Oceanologia.

[CR35] Sylwestrzak Z, Zgrundo A, Pniewski F, Lejk K, Latał A (2018) Wpływ glifosatu w postaci preparatu Roundup na zbiorowiska mikrofitobentosu Zatoki Gdańskiej – nowe doniesienia (Effect of glyphosate (Roundup® formulation) on microphytobenthic communities of the Gulf of Gdansk – new report) Technical Issues 1

[CR36] Tuji A (2000). Observation of developmental processes in loosely attached diatom (Bacillariophyceae) communities. Phycol Res.

[CR37] Van der Grinten E, Janssen APHM, Mutsert K, Barranguet C, Admiraal W (2005). Temperature- and light-dependent performance of the cyanobacterium *Leptolyngbya fiveolarum* and the diatom *Nitzschia palea* in mixed biofilms. Hydrobiologia.

[CR38] Van Leeuwe MA, Brotas V, Consalvey M, Forster RM, Gillespie D, Jesus B, Roggeveld J, Gieskes WWC (2011). Photoacclimation in microphytobenthos and the role of xanthophyll pigments. Eur J Phycol.

[CR39] Vieira S, Ribeiro L, da Silva JM, Cartaxana P (2013). Effects of short-term changes in sediment temperature on the photosynthesis of two intertidal microphytobenthos communities. Estuar Coast Shelf Sci.

[CR40] Woźniak B, Bradtke K, Darecki M, Dera J, Dudzinska-Nowak J, Dzierzbicka-Glowacka L, Ficek D, Furmanczyk K, Kowalewski M, Krezel A, Majchrowski R, Ostrowska M, Paszkuta M, Ston-Egiert J, Stramska M, Zapadka T (2011). SatBałtyk – a Baltic environmental satellite remote sensing system – an ongoing project in Poland part 1: assumptions scope and operating range. Oceanologia.

